# Barriers and Facilitators of Patient Engagement Activities to Improve Patient Safety in Healthcare Organizations: A Delphi-Based Expert Survey

**DOI:** 10.2147/RMHP.S489522

**Published:** 2024-12-19

**Authors:** Amelie Koch, Larissa Brust, Matthias Weigl

**Affiliations:** 1 Institute for Patient Safety (IfPS), University Hospital Bonn, Bonn, Germany

**Keywords:** patient involvement, patient participation, patient safety culture, healthcare, Delphi technique

## Abstract

**Purpose:**

In order to obtain sustainable healthcare, engagement of patients in patient safety improvement is vital. Drawing upon a multi-perspective approach, this study aimed to investigate perspectives of patients and healthcare professionals on key implementation factors (ie, barriers and facilitators) for effective patient engagement (PE) in healthcare organizations to improve patient safety.

**Patients and Methods:**

A two-round Delphi technique comprising semi-structured interviews and an online survey was applied to consolidate the individual perspectives of stakeholders and establish consensus on factors (expected, potential or experienced) that facilitate or mitigate successful implementation of PE in healthcare organizations (ie, all types, including hospital and outpatient medical practices). Adult, German-speaking experts in patient safety or PE (ie, with professional background or personal experience) were eligible to participate. Purposive and convenience sampling for inclusion of different healthcare professionals and patient representatives was established. Thirty-four panelists participated in data collection.

**Results:**

We identified eight key barriers and seven facilitators for effective patient engagement in healthcare organizations. Time constraints and perceived low effectiveness of patient engagement activities were deemed as most critical barriers. Supportive organizational culture, education and training opportunities, and clearly nominated responsibilities for patient feedback and engagement were rated as the most important facilitators. There were no statistically significant differences in the ratings between patient representatives and healthcare professionals.

**Conclusion:**

Our findings contribute to a deeper understanding of real-world implementation factors for effective patient engagement in healthcare organizations in Germany to improve patient safety. Our insights may further inform recommendations for future development and implementation of effective patient involvement in healthcare organizations, especially for similar countries with low levels of PE.

**Trial Registration:**

German Clinical Trials Register (ID: DRKS00031837).

## Introduction

In order to reduce harm and to achieve high-quality patient care, engagement of patients in patient safety improvement is vital. Despite continuous efforts, there are still numerous patient safety incidents worldwide, leading to increased risks of morbidity and mortality.^1^ Avoidable adverse events have been estimated to affect 2–6% of patients in hospital care worldwide.^2^,^3^ WHO’s Global Patient Safety Action Plan 2021–2023 emphasizes the importance of involving patients and their families in the future development of safe and sustainable healthcare.^4^ Insights into clinical processes and care, based on the lived experience of patients throughout their treatment process, provide a unique and valuable contribution to the prevention of errors and mitigation of risks.^5–7^ Patients can thus not only make a sustainable contribution to improving patient safety, outcomes and experience in healthcare organizations,^7–9^ but their involvement is also a moral imperative, as they directly experience care and can highlight safety issues that need to be addressed.

This active involvement of patients as partners in healthcare systems to improve the safety of care has been defined as patient engagement (PE) at a collective level.^10^,^11^ PE activities can range from simple feedback questionnaires or patient reports on safety incidents to highly participatory and collaborative activities such as joint quality improvement projects and shared leadership.^12^,^13^ Consideration of engaged patients as valuable experts allows for identification of specific needs and priorities when it comes to improving safety and care.^6^ To this end, PE is a key part of fostering patient-centred care.^14^

Although the need for PE has been repeatedly emphasized and several approaches already exist, there is a lack of systematic implementation and respective evaluation of PE.^11^ Moreover, various implementation challenges occur when healthcare leaders and institutions seek to establish PE activities, such as limited resources or lack of required abilities and knowledge (ie, health literacy) among patients.^15^ To effectively implement PE activities, it is thus crucial to conduct in-depth studies that systematically identify key implementation factors relevant to national or cultural circumstances as well as to provide meaningful recommendations for PE practices, respectively. For this purpose, it is also essential to capture experiences of all relevant stakeholders (ie, patients, healthcare professionals) to account for each party’s respective and authentic appraisals.

In contrast to established PE strategies in countries such as Canada and the UK,^16^,^17^ the involvement of patients’ perspectives on an organizational level in Germany is relatively sparse and is primarily realized through patient surveys or unsystematic assessments of patient feedback.^18–20^ Due to differences in patient safety cultures, health systems, and national policies, implementation factors already identified internationally^11^,^15^,^21–23^ cannot easily be transferred to individual countries with lower or insufficient levels of PE. To the best of our knowledge, barriers and facilitators to PE in countries with a low level of PE implementation (ie, Germany), have not been systematically explored.

The aim of this study was therefore to assess comprehensively the perspectives of patients and healthcare professionals on perceived key factors (ie, barriers and facilitators) of PE implementation at an organizational level in healthcare organizations^24^ and to consolidate their views for achieving consensus. These insights may inform future development of meaningful and evidence-based recommendations for healthcare organizations as well as consolidate key challenges within countries and healthcare systems seeking to achieve further advances in PE.

## Materials and Methods

### Study Approach

Delphi technique was applied to explore the perspectives of patients and healthcare professionals as (real-world implementation) experts for patient engagement. The Delphi method is widely used to systematically assess a broad range of different appraisals of experts in the field, consolidate individual opinions and to develop a collective consensus on a specific research question.^25^,^26^ In the first round, qualitative semi-structured interviews were conducted. Open-ended questions allowed for an exploration of individual experiences, expectations, and suggestions for patient engagement in healthcare organizations in Germany. For round 2, interview statements were condensed and presented to panelists through an online survey. With Likert scale questions, PE statements extracted from the interviews were rated by the panel experts to establish a consensus.

The ethics committee of the University of Bonn did not raise any ethical or legal concerns about the study (reference number: 091/23-EP). The study was pre-registered in the German Clinical Trials Register (ID: DRKS00031837) and can also be found in WHO’s International Clinical Trials Registry Platform. Presentation of results followed reporting guidelines for Delphi techniques.^27^

### Sampling and Recruitment

Adult, German-speaking experts in patient safety or PE were eligible to participate, ie, defined as participants with real-world and accumulated insights into patient involvement through professional or personal experiences in healthcare. Therefore, participants did not need to be experts specifically in PE implementation. Personal participation in low-level PE activities (eg, structured surveys) was also considered as personal experience in PE activities. Restricted to Germany, we used purposive and convenience sampling via multiple channels (via post, e-mail and social media) to include different healthcare professionals (eg, quality managers, physicians) as well as patients and patient representatives (also by targeting self-help groups, etc.). We thus established a sample of various stakeholder groups with respect to equal inclusion of gender and different age groups. Participants were given prior information on the study and were required to sign a written consent form. Participants were financially compensated.

### Data Collection

Two rounds of data collection were conducted from June to November 2023: In the first round, video-based individual interviews were conducted by author AK and recorded via ZoomX (Zoom Video Communications, Inc., San José, USA; Deutsche Telekom, Bonn, DE). Participant’s personal experiences were obtained as well as potential activities, corresponding expectations and influencing factors. Depending on whether the interviewees were already experts in implementing PE or had just personal experience through participation in PE activities, the interviews were based on questions starting with “Based on your professional experience…” or “Based on your personal experience…”:

1. What kind of PE activities are currently established in healthcare organizations (ie, all types, including hospitals, outpatient medical practices, pharmacies^24^) in Germany and how are these activities perceived by patients, patient representatives as well as healthcare providers?
2. What are the individual needs and expectations of stakeholders in course of PE activities?
3. What are the key implementation factors (facilitators and barriers) when introducing PE activities?
4. What would you recommend for PE in patient safety at other healthcare organizations in Germany?

Interviews ended after all questions had been answered or after a maximum of 60 minutes.

In round 2 of data collection, consolidated findings of round 1 were presented to panelists through an online survey. Statements had to be rated: for all barriers on a 7-point Likert scale ranging from 1 (“no impairment”) to 7 (“severe impairment”); for facilitators 1 (“not important”) to 7 (“very important”). Additionally, panelists rated their overall satisfaction with the current status of PE in healthcare organizations in Germany from 1 (“very weak”) to 5 (“very good”). For the online survey, the unipark.de platform (Tivian XI GmbH, Cologne, DE) was used. Each panelist received an individual invitation for the online survey and a first and second reminder if no response was submitted.

### Data Analyses

All participant data were pseudonymized (not identifiable), and all interview recordings were transcribed. Afterwards, a qualitative content analysis with an explorative approach was conducted using MAXQDA (VERBI Software GmbH, Berlin, DE).^28^,^29^ All relevant aspects stated by panelists were categorized in a two-step consensual procedure: First, text segments relevant to the study question were marked and deductively, based on the research questions, three main categories (“PE activities”; “facilitators”; “barriers”) were assigned. Two randomly selected transcripts were double-coded (by contributors AK and CR) and differences in coding were discussed by the study team. For remaining transcripts, all relevant text segments were determined, and main categories were assigned. Subsequently, the coding system with inductively created subcategories was finalized in the study team. Second, an iterative approach was applied to determine inter-rater agreement of the subcategories: one transcript was coded by two study team members (AK, CR) and Kappa coefficient (Ƙ_n_) was calculated with MAXQDA.^30^,^31^ Disagreements were discussed, and the category system was refined. This was repeated until an agreement of Ƙ_n_ = 0.84 was reached, which can be classified as “very good”.^32^ Remaining transcripts were then coded (by AK and CR) with the final category systems (Supplement material, Table A). Obtained text segments were finally paraphrased and summarized into barriers and facilitators of PE in healthcare organizations (applicable to all stakeholder perspectives).

Descriptive statistics (percent agreement, mean, standard deviation for the Likert scales) were calculated for all survey items. In addition, the consensus of panel experts regarding the prioritization of barriers and facilitators was determined. Consensus was defined as 80% or more panel experts rating scale options “5” or higher.^26^ Those items with higher scores were not rated separately against each other. To check for statistically significant differences in the ratings of patient representatives and healthcare professionals, Mann–Whitney-*U* tests were applied (with a significance level of p ≤0.05). We used SPSS Statistics 29 (IBM, Armonk, USA).

### Patient and Public Involvement Statement

Patients and their experiences were included in the planning of the study (co-design) through five unstructured discussions and interviews during planning and development stage. Additionally, one patient representative was consulted to discuss and refine the study plan. Patients and the public were not involved in the recruitment, data collection and interpretation but were part of the expert panel. Preliminary and final results were discussed in a patient-friendly language in a forum with a cancer self-help and support group.

## Results

### Expert Panel

From all over Germany, 34 experts in either patient safety or PE including non-professional and professional patient representatives as well as healthcare professionals were recruited and took part in round 1 of data collection (individual interviews). Due to the method of recruitment, the response rate for round 1 could not be determined. Response rate for round 2 was 97%, with 33 experts answering the online survey. Panel experts’ characteristics are summarized in Table 1. Most panelists reported PE experiences from hospitals.

**Table 1:** Characteristics of Panel Experts

Characteristic	Round 1 (n=34)	Round 2 (n=33)
**Age**, Mean (SD)	56.90 (10.80)	56.67 (10.87)
Background*		
Patients and patient representatives^a^	22 (16 female)	22 (16 female)
Experts in quality and risk management of hospitals^b^	7 (6 female)	7 (6 female)
Physicians^b^	3 (1 female)	2 (1 female)
Representatives of health insurance provider^b^	2 (0 female)	2 (0 female)

**Notes**: ^a^Group of patient representatives; ^b^Group of healthcare professionals; *absolute values.

**Abbreviation**: SD, standard deviation.

### Status of Patient Engagement in Germany

The overall status of patient engagement in healthcare organizations in Germany was rated with an average of 2.24 (“weak”; SD = 0.48) on a Likert scale from 1 (“very weak”) to 5 (“very good”). There were no statistically significant differences in appraisals between the groups of patient representatives (M = 2.23, SD = 0.97) and healthcare professionals (M = 2.27, SD = 0.65).

### Barriers and Facilitators of Patient Engagement

**Figure 1:**
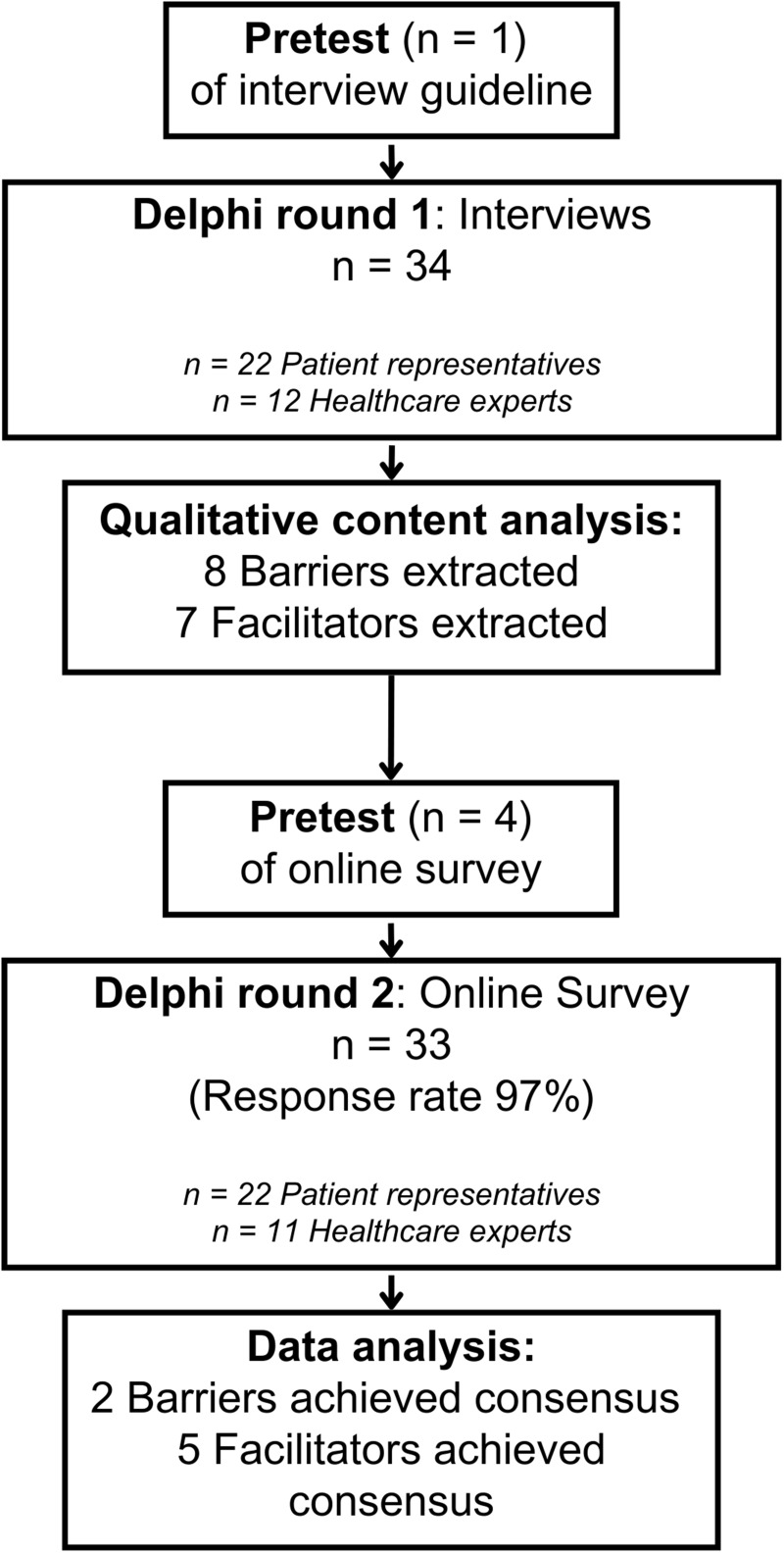
Flow chart of the Delphi process.

Overall, eight key barriers and seven facilitators of PE in healthcare organizations in Germany were identified through content analyses of experts’ answers to interview questions in round 1. Subsequently, in the online survey, two barriers and five facilitators were consensually rated as important to address when implementing meaningful PE activities and projects. Figure 1 illustrates the full Delphi process and the numbers of barriers and facilitators obtained.

Table 2 depicts panelists’ ratings of identified barriers (round 2 survey). High ratings were achieved for time constraints as the most critical barrier to PE in Germany. It was reported that healthcare providers already struggle to manage their high workload and that there is hardly time left for PE activities. On patient’s side, participation in improvement processes is predominantly voluntary and non-paid, with potential for conflicts between patients’ employment and their additional involvement in time-consuming PE activities. The second most critical barrier to the implementation and long-term establishment of PE was perceived low effectiveness of PE activities. For example, healthcare organizations have widely implemented patient feedback questionnaires, but results are rarely analyzed in detail and often lack practical impact on patient safety improvements, leading to frustration for all those involved.

**Table 2:** Rating and Consensus on Barriers of Patient Engagement Activities in Healthcare Organizations in Germany

Barriers	Agreement^a^ (%)	Overall	Patients	Healthcare Professionals
		Mean (SD)		
Time constraints (eg patients and staff in healthcare organizations lack sufficient time)	**84.4**	5.97 (1.21)	6.23 (0.97)	5.45 (1.44)
Perceived low effectiveness (eg patient feedback is obtained but not utilized)	**81.2**	5.84 (1.30)	5.61 (1.40)	6.27 (0.96)
Insufficient organizational structures (eg no responsible staff members for PE activities)	78.1	5.47 (1.54)	5.42 (1.43	5.55 (1.72)
Employee’s fear of negative consequences (eg social distancing following criticism)	65.6	5.50 (1.32)	)5.57 (1.18)	5.36 (1.55)
Interpersonal barriers (eg patients do not want to offend)	62.5	5.21 (1.65)	5.14 (1.52)	5.36 (1.87)
Patient’s fear of negative consequences for their own treatment	53.1	5.09 (1.74)	4.95 (1.70)	5.36 (1.77)
Financial constraints (eg no hospital budgets for professional questionnaire analyses)	53.1	4.91 (1.81)	4.62 (1.68)	5.45 (1.92)
Low quality of patient feedback (eg feedback is not applicable)	40.6	4.25 (1.58)	4.24 (1.41)	4.27 (1.86)

**Notes**: ^a^bold, if consensus achieved was >80% of panelists rated 5 or higher on 7-point Likert scale (1 “no impairment” to 7 “severe impairment”).

For six barriers, consensus was not obtained among panelists (cf Table 2). Nevertheless, all barriers were on average rated as rather critical (>4 points on the 7-point Likert scale).

All obtained facilitators, as well as achieved consensus, and ratings are listed in Table 3. The highest consensus for facilitators of PE in Germany was achieved for supportive organizational culture. Panelists rated a culture of mutual support, trust, and appreciation with opportunities for critical feedback within a healthcare organization to be highly relevant for success. Second and third most important facilitators were education and training opportunities (ie, subject-specific knowledge and social skills for effective collaboration) for all stakeholders and establishment of official units in healthcare organizations for patient feedback and engagement. According to the panelist, clearly defined internal responsibilities for PE, effective information channels and public relations work are essential to ensure patient participation. Additionally, integration of PE activities into existing workflows and structures of healthcare organizations (eg, embedding PE in quality management or hospital discharge processes) would foster the implementation process, save costs and increase acceptance. Networking and exchange of experience and expertise between stakeholders and across organizations was also highly rated, particularly due to the very limited experience with PE activities in Germany to date. For two facilitators, panel expert consensus was not achieved. Still, high average ratings (all >5 on 7-point Likert scale) indicate the importance of these aspects (cf Table 3).

**Table 3:** Rating and Consensus on Facilitators of Patient Engagement Activities in Healthcare Organizations in Germany

Facilitators	Agreement^a^ (%)	Overall	Patients	Healthcare Professionals
		Mean (SD)		
Supportive organizational culture (eg critical feedback is encouraged)	**96.9**	6.75 (0.66)	6.62 (0.79)	7.00 (0.00)
Subject-specific education and training opportunities for patients and facility staff	**96.8**	6.48 (0.91)	6.71 (0.54)	6.00 (1.26)
Establishment of an official unit in healthcare organizations for patient feedback and engagement (ie clearly nominated responsibilities)	**93.8**	6.69 (0.77)	6.57 (0.90)	6.91 (0.29)
Integration of (new) PE activities into existing organizational workflows and structures (eg embedding PFE in quality management or hospital discharge processes)	**93.8**	6.41 (1.09)	6.29 (1.24)	6.64 (0.64)
Networking and exchange of experience and expertise	**84.4**	6.19 (1.18)	6.28 (1.05)	5.82 (1.34)
Possibility of anonymous feedback/participation	75.0	5.63 (1.47)	5.38 (1.46)	6.09 (1.38)
Compensation for expenses (eg financial compensation for patients’ expenses)	62.5	5.06 (1.75)	5.57 (1.40)	4.09 (1.93)

**Notes**: ^a^bold, if consensus achieved was >80% of panelists rated 5 or higher on 7-point Likert scale (1 “no impairment” to 7 “severe impairment”).

No statistically significant disagreements could be found in the ratings of barriers and facilitators between the groups of patient representatives and healthcare professionals.

## Discussion

Although patient engagement is conceived as a key strategy to foster safe care practices, our knowledge base on implementation factors for successful activities and approaches at an organizational level in Germany is still limited. In a Delphi-based survey comprising multiple stakeholder perspectives, we gathered valuable insights on patients’ and healthcare professionals’ opinions on essential PE implementation factors in Germany. We were also able to confirm and corroborate existing PE implementation knowledge from other national and healthcare settings. Moreover, we did not observe any significant discrepancies between evaluations of patient representatives and healthcare professionals. Our findings provide several important contributions to the current research based on implementation of PE activities in patient safety and sustainable healthcare improvement.

Although the importance of PE has been repeatedly emphasized,^4^,^33^ its overall status in Germany was perceived as insufficient – by both patients and healthcare professionals. Our expert-based findings propose key factors for successful implementation: Time constraints and the perceived low effectiveness of PE activities were unequivocally rated as the most challenging barriers. A supportive organizational culture encouraging mutual trust and respect, focused PE education and training opportunities, and the nomination of clear responsibilities or official units within healthcare organizations for patient feedback and engagement (ie, PE managers) achieved high agreement and were thus deemed to be the most important facilitators.

Several of the implementation factors we identified resonate well with previous results of a systematic review of barriers and facilitators of PE at the individual level:^15^ ie, a supportive and trustful relationship between patients and providers and high workloads among providers. In addition, education and training opportunities for PE stakeholders have been identified as key facilitators, not only in this study but also in previous research pointing out the role of missing individual knowledge and lack of training resources.^8^,^11^,^15^,^21^ Competencies such as health literacy, patient safety and communication skills could be targeted through tailored educational approaches. Concerns and fears of negative consequences due to active participation in PE activities have also been suggested in previous studies.^15^,^34^,^35^ Nonetheless, these reservations previously tended to be reported from patients and their representatives, rather than healthcare professionals, as it was the case in the present study. However, a supportive organizational culture is key to overcome this barrier.^11^,^15^,^34^

Interestingly, other studies have identified challenges in recruiting patients for the role of patient representatives or patient advocates as one of the barriers,^11^,^21^,^36^ which was not the case in this study, except for compensation for expenses. We assume that due to the limited number of PE activities in Germany, recruitment is currently not the foremost problem. In addition, these different findings may be due to our participants who did not fail to conduct PE activities due to recruitment, or who were self-motivated to participate in PE activities. Influences of patient’s demographic characteristics were not relevant in this study but have been discussed in previous studies.^15^,^34^,^37^ In contrast to the existing literature, our study identified low quality of patient feedback as an important barrier to PE. From the perspective of healthcare professionals, poor design and contents of patient feedback measures limit the valid identification of needs for improvement and learning. This results in poor usability and perceived low effectiveness of PE activities. However, perceived inefficiencies could be attributed to various reasons such as when patient-reported experiences are rarely analyzed in detail, results lack actionable findings and practical impact, or healthcare institution lack ability to act upon PE. Future research should address how hospitals can elicit relevant feedback from PE actors as well as scrutinize how opportunities for anonymous reporting align with more participative forms of PE on organizational levels.^15^ In some cases, however, patients may not want to be anonymized or silenced if they wish to be heard as a person.

### Strengths and Weaknesses of the Study

We followed a step-wise approach and included a diverse expert panel with a broad range of experience, as well as healthcare professionals across various professions and specialties. We received a very high response rate for the second round, which strengthens the internal and external validity of our results.^38^ By converging qualitative and quantitative data (iterative approach) and considering different perspectives with a high level of agreement between patients and health professionals, identified factors for PE implementation were well confirmed.^39^ Since the finally generated number of elicited PE barriers and facilitators is limited, they might easily be addressed, adopted and considered by health care institutions. Finally, our study has a high practical relevance for advancing systematic implementation of PE activities to promote patient safety, especially in healthcare settings with currently low adoption of PE.

Nonetheless, our results should be interpreted in the light of several limitations: First, external validity may be confined due to purposive selection of panelists, sample size, and its restriction to a national context. However, our selection approach established a diversity of key stakeholders across different healthcare sectors. Second, data is based on individual statements and subjective appraisals. Third, the recommendations (see below) were in the end not separately approved by surveyed participants (eg via focus group). Furthermore, since there is a lack of highly participatory PE in Germany, panelists may not have relied only on their first-hand experiences in PE. As a result, some of the identified barriers and facilitators might be deemed potentially relevant but have not necessarily been experienced in the real-world. Since we focused on implementation factors in healthcare organizations, results may mainly refer to hospitals. Transfer to other healthcare sectors (ie, outpatient care) needs to be scrutinized in depth in the future. Finally, few of the suggested implementation challenges (eg, establishment of intra-institutional official units for PE) might be especially relevant to countries with a low level of PE activities and strongly depend on regulatory context, national policies and guidelines. However, we strongly believe that institutions with advanced PE activities may also benefit from bearing in mind the reported PE implementation factors.

### Implications for Healthcare Practice and Research

Healthcare systems worldwide struggle with various demands such as increasing load of complex and chronic cases, staff shortages, and constraints in resources.^40^,^41^ At the same time, involving patients and representatives in promoting high-quality care, equity and patient safety remains a challenge.^5^,^11^,^21^,^34^,^36^ To this end, our findings might help to improve existing PE collaborations or to implement new PE activities successfully in the future, especially for those countries where the involvement of patients’ perspective at the organizational level has been sporadic and unsystematic.^18^,^19^ There is a strong moral argument for PE in general and in patient safety specifically to promote harm-free, accountable, and high-quality healthcare services. Our findings suggest several future avenues for healthcare institutions to enhance patient engagement. Among others, this may include (1) promoting an organizational culture that values patients’ feedback and engagement; (2) providing educational resources and training opportunities for patients and staff should be provided to enhance knowledge, skills and competencies relevant for PE; (3) defining and communicating a comprehensive strategy, with clear organizational responsibilities for sustainable PE; (4) supporting networking opportunities (within and across organizations) for all stakeholders to share, exchange and discuss existing PE experiences and strategies; and (5) lastly, seeking opportunities to evaluate PE activities, also with attention to impact in everyday care practice.

Future research should particularly study factors that initiate and promote PE activities in various healthcare settings, systematically compare different types of PE interventions, as well as pay particular attention to processes in the course of successful and sustainable PE practices. Further insights into the implementation challenges and associated impacts on healthcare outcomes of PE activities are needed. This might also help to overcome the identified barriers of perceived low effectiveness of PE and limited time resources.^11^,^42^ Concerning further facilitators, future research should elicit what kind of educational resources and training approaches could effectively foster PE; and how healthcare organizations and leaders can support patients and their representatives to overcome the reported barriers. Furthermore, comprehensive empirical surveys of the current state of PE to promote patient safety in Europe are missing. Future investigations of PE activities should also capture different care sectors as well as patient journey across sectors, particularly in specific safety-critical care processes such as for example discharge after inpatient care.^43^,^44^

## Conclusion

This study contributes to current knowledge by providing valuable insights into key factors of PE implementation in health care institutions in Germany as perceived by patient representatives and healthcare professionals. Our findings may inform future PE strategies and activities, to foster a more systematic and thorough inclusion of patients’ perspectives to promote patient safety and sustainable quality of care by enhancing safety practices, optimizing resource use, and supporting system adaptability in a way that is responsive to patients’ needs. Our findings may also help other countries with low levels of PE implementation.

## Data Availability

Data underlying this study are available within the manuscript or supplementary material, to the extent permitted by data protection legislation. Further data (in German language) can be made available by the corresponding author on reasoned request.
